# The Problem of the Independent Poor

**Published:** 1916-05

**Authors:** 


**A Monthly Review of Medicine and Surgery**

EDITOR AND PUBLISHER, DR. A. L. BENEDICT, 228 Summer St., corner of Elmwood Ave., Buffalo, (Address for all communications. Please make personal and telephone calls before 1 P. M.)

Third, in age, on the western continent. Independent, but supports professional organization. News limited mainly to Western N. Y. and Penn.

Subscription $2.00 a year; $3.00 for two years in advance. Changes and errors in address or failure or duplication of delivery, should be reported immediately.

Yearly Volume 71 MAY, 1916 No. 10

### The Problem of the Independent Poor.

Our attention has been called to this matter partly by various articles in journals devoted mainly to hospital management. These, for the standpoint of the hospital, very properly insist on adequate charges for ward and private room services. Counting necessary expenses for thorough care in various directions, it appears that the actual, average cost, of a ward patient is at least a dollar a day, and of a patient in an unassuming private room, about double this amount. In some instances, the cost runs far above the average. For example, may be cited a ward case in which an interne calculated that the single item of iodoform (this was some years ago) came to more than the hospital was receiving for the entire care of the patient. Co-operation, on a large scale, does not materially reduce the cost, any more than the tax rate in a large city falls below that of a village. To a considerable degree, the cost of hospital service is high because of what is sometimes termed extravagance, meaning the routine application of rather more elaborate laboratory methods than would be employed in private attendance, the use of new drugs, liberal expenditure of time of assistants, sacrifice of petty economies for the convenience and pride of attendants, etc. But, it must be admitted that all these extravagancies amount, in the aggregate to a very meagre honorarium for professional services donated, and that they are justified, not only on this ground, but because the hospital exists not simply to care for the existing patient, but to establish high standards of professional work and to lead to further improvements of both the science and the art of medicine, in the interest of future patients, in and out of hospitals.

The economic problem is simple enough for the rich and equally simple for the pauper, using the latter term either in its extreme meaning, or as applied to the poor person who fully expects aid in an emergency. Here may be mentioned the peculiar popular attitude of perfect willingness to accept medical charity in any private form, but which regards as a disgrace the acceptance of the services of public physicians, paid by taxpayers to care for the poor, or of the shelter of an institution similarly provided. To our mind, whatever disgrace accompanies the acceptance of public medical care, ought to be manifested in exactly the opposite direction. For instance, to put the matter in personal form, we have for years contributed in the form of taxes, to pay the salaries of city physicians and of county hospital, as well as for the maintenance of city patients in various hospitals. Why should we not, in case of need, accept these services for which we have paid, pro rata, in advance, in the same spirit that we would accept insurance on which we had paid premiums? And, even if the individual has not paid direct taxes, he has contributed indirectly to the accumulation of wealth on which taxes are paid, and he may properly regard himself as insured in the same way, as a member of the community at large.

But let us consider the economic problem as it applies to the majority of independent persons, not in any sense of the pauper class, and who are forced by general opinion to maintain a scale of medical attendance and care in sickness and injury, far beyond that of the charity cases, deserving or merely helpless and demanding service on humanitarian grounds, though in this position by reason of definite economic sins of omission and commission. We speak glibly of the submerged tenth of the population. Probably at least 20% is submerged in the sense of being charity patients in any sickness which is absolutely disabling and of more than very brief duration. On the other hand, not more than 10% and probably not over 5% of the entire population is able to pay, without serious sacrifice, the exhaustion of savings or the incurring of protracted debt, for the medical and hospital services involved in a fairly severe illness, obstetric case or capital operation, graded on the basis of minimum private room charges.

In other words, we estimate that approximately 70% of the population has a family income of somewhere from $1000 to $2500 and that, under present conditions of scale of living and expense for routine necessities, the emergent expense of a disabling medical or surgical condition, amounts to a catastrophe.

It is an axiom of sociology that any emergency which leads to temporary dependence upon charity of any form, is disastrous, because it tends to enfeeble self respect, and to lead to permanent pauperization. And it is obvious that any emergency which leads to more than transient inconvenience and financial crippling is to be avoided if possible. This is especially true of emergencies involving physical disablement because, either in the individual himself or some one directly involved in his financial recuperative powers, such recuperation is rendered doubly difficult.

The worst feature of this economic problem is that it seems impossible, under existing conditions, either to reduce the expense of hospital care to the ability of the average citizen, or to elevate the earning power of the latter to the necessary scale of hospital and professional charges. In discussing all such problems it must be remembered that the most extreme application of socialistic principles would result only in bringing everyone to a financial basis which no one would consider worth while, and that this equalization of wealth would be merely temporary.

There are, some factors of the problem that contain a hint as to its solution. First, most hospitals that are in any sense public, are already supported to some extent by public taxation. Secondly private benefactions are, in an informal and voluntary way, graded according to the principles of the income-tax, involving the broader principle that greater wealth than the average maximum is derived from the community at large and is fairly subject to taxation for the benefit of every member of the comunity. Thirdly, the Catholic Church maintains and, so far as possible, inforces, a standard of benevolence that often seems cruelly high to Protestants but is justified when one considers the varied forms of benevolent institutions conducted for the benefit of the persons contributing, whenever they have real need of charity at their hands. Fourthly, the principle of insurance against the need of professional service and institutional care is already in operation along many private lines, though universally under such conditions as to receive the denunciation of the medical profession. This denunciation is based on two well established grounds: unfairness in selection of medical attendant, and inadequacy of the insurance fee, whatever it may be called, these two factors resulting in the third evil that the care furnished is usually of low grade.

The principle of insuring against emergencies, individually disastrous, but by the law of chance representing an average expense of comparatively small amount, is fully established along various lines. It would seem that a careful estimate of the chances of sickness and the like would render feasible an insurance of hospital care in any emergency, on a basis that would be equable alike to hospital and patient. There are good reasons, both discernible in advance and demonstrated by English experience against extending this insurance to cover actual professional services. Briefly stated, these are the practical difficulties of providing for free choice of attendant, particularly when special skill is required, and for covering the proper graduations of fees according to professional skill, prestige and the like. As it has been the established custom of the medical profession for many generations, to grade its fees according to the means of the patient and to consult his convenience as to the time of payment, the apparent solution of only half of the problem, really solves the whole problem in its most exigent form.

